# Is targeted next generation sequencing for drug-resistant tuberculosis feasible in a high-burden setting: a qualitative study from Indonesia

**DOI:** 10.1136/bmjopen-2026-117319

**Published:** 2026-08-11

**Authors:** Salma N. Annisa, Almira Alifia, Alamanda N. Larasmanah, Nurlaila S. Ramadhani, Silvi Indriani, Arto Yuwono Soeroto, Reinout van Crevel, Philip C. Hill, Bony Wiem Lestari

**Affiliations:** 1 Research Center for Care and Control of Infectious Diseases, Universitas Padjadjaran, Bandung, Indonesia; 2 Department of Internal Medicine, Division of Respirology and Critical Care Medicine, Hasan Sadikin General Hospital, Bandung, Indonesia; 3 Faculty of Medicine, Universitas Padjadjaran, Bandung, Indonesia; 4 Department of Internal Medicine, Radboud Community for Infectious Diseases (RCI), Radboud University Medical Center, Nijmegen, Netherlands; 5 Faculty of Medicine and Dentistry, Blizard Institute, Queen Mary University of London, London, UK; 6 Centre for International Health, Division of Health Sciences, University of Otago, Dunedin, New Zealand; 7 Faculty of Medicine, Department of Public Health, Universitas Padjadjaran, Bandung, Indonesia

**Keywords:** Tuberculosis, Health Services, Molecular diagnostics, Indonesia

## Abstract

**Abstract:**

**Objectives:**

Targeted next-generation sequencing (tNGS) offers promise in the rapid detection of drug-resistant tuberculosis (DR-TB) directly from sputum. While pilot studies on tNGS are emerging, there is limited empirical qualitative evidence on tNGS implementation. This study, therefore, aimed to explore the experiences of tNGS programmatic implementation by clinicians, laboratory technicians and policymakers to identify perceived key operational barriers and enablers.

**Design:**

An exploratory qualitative study employing inductive thematic analysis. Data were collected via semi-structured interviews and focus group discussion. Analysis was guided by the theoretical framework of acceptance.

**Setting:**

This study was conducted between March and October 2025 as part of a broader implementation study evaluating tNGS potential programmatic integration in West Java, Indonesia. Participants were recruited from a DR-TB reference laboratory and a tertiary care hospital.

**Participants:**

A purposive sample of 18 tNGS users participated, including clinicians (n=7), laboratory technicians (n=9) and policymakers (n=2).

**Results:**

Analysis revealed a dual challenge for tNGS implementation: technical-operational complexity and unguided clinical utility. Technical barriers include laboratory workflow optimisation and the need for highly skilled technicians for routine operation. From a clinical point of view, lack of local tNGS guidelines for clinicians and absence of local evidence such as cost-effectiveness and diagnostic performance at the policy levels hampered clinical adoption. Several enablers for tNGS implementation were identified, namely technicians with prior sequencing experience, perceived clinical value for complicated cases and advocacy within professional networks.

**Conclusion:**

Successful scale-up of tNGS for DR-TB in high-burden settings requires a coordinated multisectoral strategy. Generating local evidence on robust laboratory data for technical guidelines, clinical utility and cost-effectiveness are warranted to support tNGS adoption. This evidence can then be integrated into the national tNGS roll-out strategy to enable sustainable and nationwide adoption of tNGS.

**STRENGTHS AND LIMITATIONS OF THIS STUDY**

- This study uses an exploratory design involving multistakeholder perspectives, appropriate and relevant to the matter being investigated, as targeted next-generation sequencing (tNGS) is new to Indonesia and requires different types of expertise for its use.
- By purposively sampling informants with direct, hands-on exposure to tNGS (either operating the platform, interpreting clinical results or shaping the policy landscape), the study ensures that the captured insights are grounded in concrete operational realities rather than hypothetical assumptions.
- Because recruitment was restricted to actors with prior tNGS exposure, the potentially broader implementation barriers perceived by actors without similar exposure could not be captured.
- The study focused on a single province and its reference laboratory that uses a specific technology platform, and hence the operational nuances identified here may differ from other settings.

## Introduction

 Drug-resistant tuberculosis (DR-TB) continues to rise worldwide. Multiple efforts have been made to control DR-TB, including innovations in DR-TB diagnostic tools. Currently, the gold standard to diagnose DR-TB is phenotypic drug susceptibility test (pDST), which is culture-dependent and takes weeks to generate results, creating delay to appropriate treatment that is crucial for patients with DR-TB. Molecular WHO-recommended rapid tests such as Xpert MTB/RIF Ultra (for rifampicin resistance) and Xpert MTB/XDR (for second-line drug resistance) have been reported to significantly reduce the waiting time for DR-TB diagnosis; however, their coverage is limited to several prioritised drugs only.^1^

Targeted next-generation sequencing (tNGS) has recently emerged as a potential transformative diagnostic tool for DR-TB, enabling a relatively rapid detection of resistance-associated mutations towards a wide range of anti-tuberculosis drugs.^2^ tNGS could help overcome the main limitation of pDST turnaround time.^3^ The WHO has issued a recommendation to use tNGS as a follow-on test for initial DR-TB diagnosis.^1^

Indonesia is considered a high-burden TB setting with an estimated incidence of 382 per 100 000 people in 2024, and a recorded 12 512 DR-TB cases in 2025.^4 5^ This situation puts Indonesia as a critical setting for exploring the implementation of tNGS. Control of DR-TB in Indonesia faces challenges such as patient attrition, diagnostic and treatment delays.^6^ Currently, the implementation of tNGS for DR-TB in Indonesia is still limited. At the national level several sites are involved for genomic surveillance, and there is a recent implementation study for tNGS use in West Java.

Current research on tNGS for DR-TB has primarily focused on its technical performance for DR-TB diagnosis.^7–10^ Beyond laboratory-based performance studies, a significant gap exists in understanding the implementation of tNGS as part of a routine programmatic service.^11^ There have been qualitative investigations comparing the perceived benefits and barriers of NGS-based TB diagnostics in Madagascar and Canada, and an assessment of healthcare providers’ knowledge and attitudes toward tNGS utilisation in Eswatini.^12 13^ While these studies provide foundational insights, they offer limited data on user experiences of tNGS in day-to-day clinical and laboratory practice, and how policymakers navigate its programmatic adoption.

A recently completed tNGS implementation study in West Java provides an opportunity to gather insights on tNGS use in a high-burden setting in Indonesia.^4^ Therefore, this study aimed to explore the experiences of early adopters including clinicians, laboratory technicians and policymakers, with the implementation of tNGS for DR-TB. Additionally, we also aimed to identify key operational barriers and enablers for potential tNGS scale-up and national adoption in Indonesia.

## Methods

This was an explorative qualitative study, adopting the theoretical framework of acceptability (TFA) to explore tNGS multifaceted acceptability among stakeholders. According to the TFA, acceptability is not a singular attribute, but a multidimensional construct shaped by the subjective experiences of those delivering or receiving an intervention.^14 15^

### Study setting

This study was conducted as part of an early tNGS implementation study in West Java, Indonesia.^4 16^ West Java is among the most populated provinces in Indonesia, with a population of 50 million people in 2025, which was around 17% of the national population. The province has the highest tuberculosis case load in Indonesia with an estimated number of 234 710 TB cases and reported 3147 DR-TB notifications in 2024.^17^

In West Java, DR-TB services are provided through a network of 40 hospitals and three referral laboratories for culture and phenotypic drug-susceptibility testing. tNGS use for DR-TB is not yet included in Indonesia’s national diagnostic algorithm. As part of the tNGS implementation study in West Java, tNGS service was available during the study period at the West Java Provincial Health Laboratory using Deeplex-MycTB with iSeq100 or MiSeq i100Plus sequencers.

### Patient and public involvement

Patients and the general public were not involved in the study.

### Data collection and analysis

This study is reported in accordance with the ‘Consolidated Criteria for Reporting Qualitative Research’ (COREQ) checklist. The completed checklist is available as supplementary material (online supplemental file 1). All procedures and data collection instruments were in accordance with the protocol (online supplemental file 2).

The study was conducted between March and October 2025, employing semi-structured interviews and a focus group discussion (FGD). Participants were recruited via purposive sampling with snowballing elements. We selected potential informants defined as key experts including clinicians, laboratory personnel and government officials. Participants were included if they met at least one of the following criteria: (1) practicing clinicians actively engaged in the clinical management and treatment decision-making of patients with DR-TB; (2) laboratory personnel with direct experience managing and/or operating tNGS workflow; or (3) regional or national policymakers actively involved in early-stage tNGS pilot initiatives. Conversely, individuals were excluded from participation if they were directly involved in the design, administration or execution of this tNGS implementation study itself. The pool of eligible participants was small as tNGS implementation for DR-TB in Indonesia is very limited, especially outside of West Java.

Potential participants were contacted with a formal invitation letter; all accepted and provided written informed consent. A total of 14 interviews and two FGDs were conducted in Indonesian and were mostly in-person at participants’ workplaces, except for one online interview with a former National Tuberculosis Control Programme (NTP) official due to geographical constraints. Participants were provided with a souvenir as a token of appreciation.

Data collection was led by SNA, a female qualitative researcher with a master’s degree and 5 years of experience in health research. To enhance analytical rigour and mitigate interviewer bias, each session was co-facilitated by a second researcher knowledgeable in public health laboratory management (ANL, AA) with epidemiological and public health expertise, respectively. No prior relationship existed between the researchers and participants. Each session began with introductions and a detailed explanation of the study’s purpose. Interview and discussion guides were not shared with participants in advance of the interviews. Due to the limited pool of eligible participants, the interview and FGD guides were not formally piloted to preserve sample size for the primary analysis. Instead, instrument validity was established through being reviewed and refined by an interdisciplinary consensus panel of senior clinicians, microbiologist and social scientist.

One FGD was held with laboratory technicians to capture interactive group dynamics, moderated by SNA and assisted by ANL and two other research assistants. A follow-up FGD session was conducted with two original participants to clarify points and enrich the data.

Sample size was determined by the principle of thematic saturation. Data collection continued until saturation was reached. We identified the saturation point during the analysis of the final two interviews. All interviews and discussions were audio-recorded and transcribed verbatim for subsequent analysis. SNA led the transcription process with one research assistant, NSR. Thematic analysis followed a hybrid approach combining inductive and deductive elements. First, SNA independently conducted inductive open coding from the transcript with Atlas.ti software (V.9.20.0). Second, SNA and AA systematically restructured and mapped emergent codes onto the healthcare acceptability framework (see online supplemental file 3). Member checking was performed by re-contacting several clinicians via a messaging application to confirm preliminary findings/themes. Data triangulation was employed to ensure interpretive rigour, using a diverse dataset comprising interview transcripts, participant observation notes and field notes. Key themes were converged on through iterative discussions among researchers SNA, AA and BWL.

## Results

### Characteristics of study participants

14 individuals were interviewed, including seven clinicians from DR-TB treatment centres, a former laboratory vocal point at the NTP, a provincial tuberculosis supervisor, a biomolecular laboratory manager and four tNGS laboratory technicians. Additionally, four laboratory members participated in an FGD; three worked as tNGS operators and one handled pre-analytical steps. Detailed participant characteristics are presented in table 1.

**Table 1:** Characteristics of study participants (N=18)

Characteristic	Clinicians (n=7)	Laboratory technicians (n=9)	Policymakers (n=2)
Background of study participants			
Specialist (pulmonologist or internist)	7	–	–
General practitioner	–	–	1
Public health specialist	–	–	1
Biomolecular scientist	–	8	–
Medical laboratory technologist	–	1	–
Age, in years (median, IQR)	46 (46–49)	27 (24–32)	52 (52.3–52.8)
Gender			
Male	2	3	–
Female	5	6	2
Type of data collection			
In-depth interviews	7	5	2
Focus group discussion	–	2	–
Duration of the interview/FGD (median, IQR), in minutes			
In-depth interviews	60 (38–72)	75 (70–76)	96 (91–101)
Focus group discussion	–	85 (66.5–103)	–
Experience in DR-TB management, in years (median, IQR)	12.3 (11.2–12.7)	1.6 (1.1–9.7)	5.7 (4.6–6.8)

DR-TB, drug-resistant tuberculosis; FGD, focus group discussion.

### User perceptions of barriers and enablers of tNGS implementation

**Figure 1:**
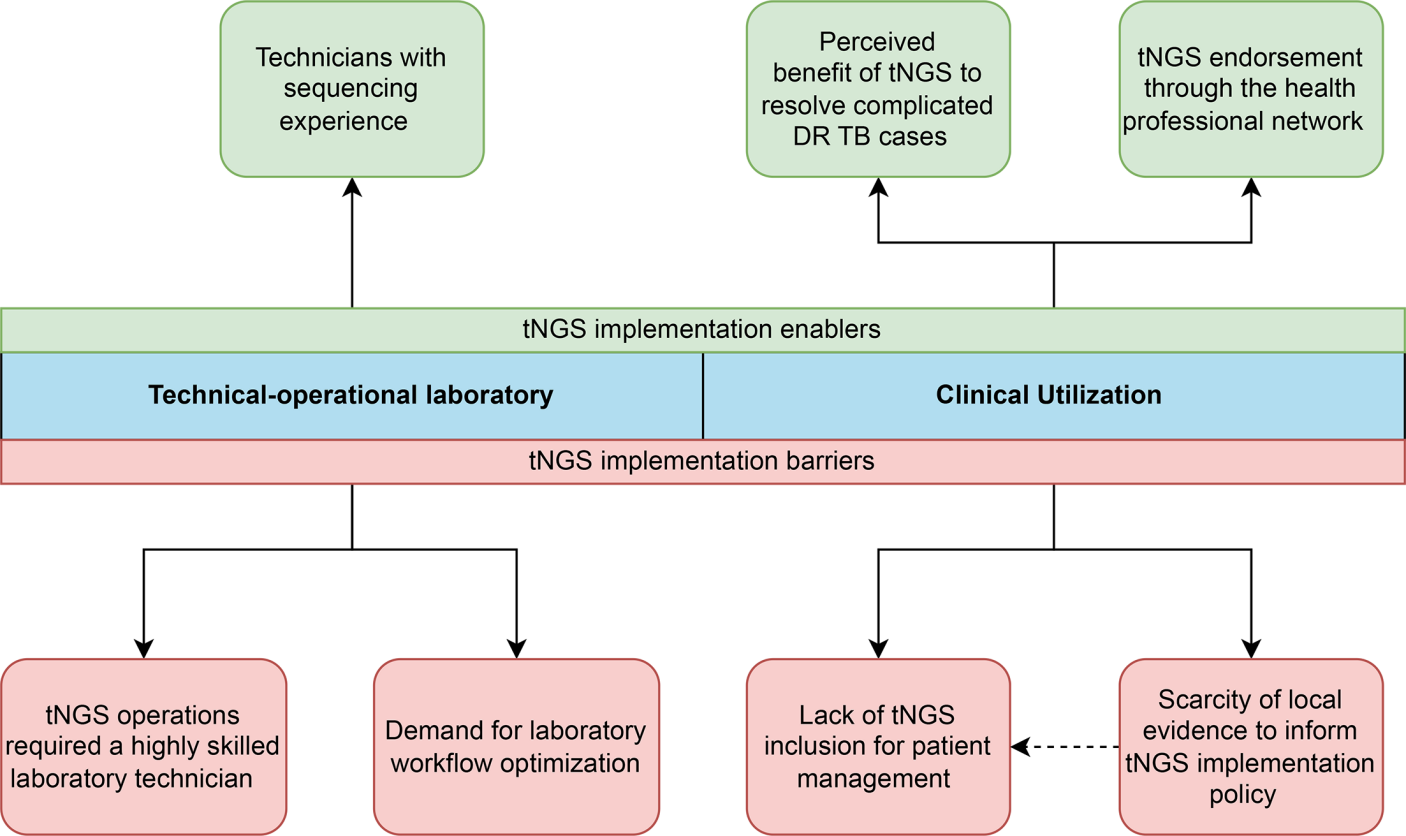
Enablers and barriers associated with tNGS implementation for DR-TB diagnosis. A thematic map synthesising findings from interviews and a focus group discussion with clinicians, laboratory technicians and policymakers in West Java, Indonesia. Key enablers (technicians with sequencing experience, perceived clinical benefit for complicated cases, professional network endorsement) and barriers (need for highly skilled technicians, laboratory workflow optimisation, lack of clinical guidelines, scarcity of local policy evidence) are categorised within the two overarching domains of technical-operational factors and clinical utility factors. DR-TB, drug-resistant tuberculosis; tNGS, targeted next-generation sequencing.

Thematic analysis revealed distinct user perceptions of tNGS, structured around two core dimensions of implementation: technical-operational laboratory processes and clinical utilisation. The associated barriers and enablers within each dimension are synthesised in figure 1, with a detailed exposition of supporting themes and representative quotes in the supplementary material (online supplemental file 4).

## tNGS implementation from laboratory perspective

### Technicians with sequencing experience

Laboratory technicians with prior sequencing experience are key enablers for facilitating effective tNGS laboratory operation. Four out of nine laboratory technicians gained their skills on sequencing when they worked in a COVID-19 referral laboratory during the pandemic. Through their experiences these individuals obtained the capacity to reliably execute technically demanding workflows like library preparation and to troubleshoot issues that arose.

I think the most important is experience. It’s risky (to rely on inexperienced technicians). For example, you need to understand the theory behind tNGS, like how PCR works, how library preparation works, and what single-stranded DNA is. Then, how does (nucleic acids) amplification work? These things require knowledge and experience. If they don’t have these, then it will be a bit difficult. They could get stuck. (LM 1, laboratory manager, individual interview)

Furthermore, they served as trainers of trainers for newly recruited staff, compensating for the lack of formal initial training. This established a mentor-apprentice model that was essential for sustaining operations.

Speaking from my experience… when I first started, I genuinely didn’t know anything about tNGS, so I just learned from [other tNGS technician]. So, I mostly learned from her and read on my own. (TO 4, tNGS operator, FGD)

### tNGS operations required a highly skilled laboratory technician

The informants highlighted the need for highly skilled technicians in sequencing particularly for the library preparation step. This step involves meticulous, manual tasks like using micropipette and manually calculating dilutions. Cumulatively, these detailed tasks are not only demanding but also lengthy, often consuming more than a day.

Human resource competency is extremely crucial. It’s important because not all lab technicians are accustomed to handling such highly detailed work like this (micropipette and manual calculations), where the process can take more than a day. That experience and the endurance to carry out such lengthy procedures are important factors in the lab. (TO 9, tNGS operator, individual interview)

The need for skilled technicians was emphasised to mitigate the risk of costly batch failure. As one laboratory manager mentioned, early-stage issues such as undetected inhibitors during PCR amplification could carry through to the pooling stage, degrading overall sequencing quality and compromising final results.

Why experienced technicians matter? For instance, in the tNGS process, there’s the PCR step where DNA gets amplified and is increased in concentration. But if there are inhibitors in the sample and the technicians don’t realize or don’t know and just like ‘oh, this actually got amplified’ but the inhibitors are still present later during the pooling step, it will affect everything. It can degrade the quality of the sequencing. (LM 1, laboratory manager, individual interview)

Some laboratory informants also expressed that recruitment of technicians with biomolecular capacity is hampered by local standards for recruitment in Indonesia. This barrier is perceived to be a bottleneck for further tNGS upscaling.

For it (tNGS) to become a routine program, in my opinion, the changes would have to be very large-scale. The change even starts with the recruitment system. It has to be fixed first. So, we have to open recruitment for those with a molecular biology background. But several labs can’t just recruit technicians freely on demand. (TO 1, tNGS operator, individual interview)

### Demand for laboratory workflow optimisation

Another barrier reported by laboratory technicians was the need to optimise the workflow. They reported that successful sequencing most often requires in-house adaptation of laboratory protocols. A key example was the occurrence of PCR inhibitors. To resolve this, the laboratory technicians incorporated additional drying and centrifugation steps. Although this optimisation was anticipated, the additional procedures incurred a significant time and financial burden due to extra logistics and consumables for sample retesting. Technicians still opted to do such optimisation considering sequencing failures as direct threat to the service itself.

Back then, we had no way to see the inhibitor. The only option was to test the reagents first… to find which sequencing reagent produced the best result. However, conducting that trial requires both money and time. That’s why identifying the inhibitor was quite (technically and financially) challenging. (LM 1, laboratory manager, individual interview)

This optimization issue cannot be ignored. Considering that tNGS is complex and the unit cost is high, errors that affect results like this should be a concern for sustainability. (TO 1, tNGS Operator, individual interview).

## tNGS implementation from clinical utility aspect

### Perceived benefit of tNGS to resolve complicated DR-TB cases

Clinicians highlighted the potential benefit of tNGS for solving complex cases such as patients with persistent culture positivity or discrepant results from phenotypic or rapid molecular testing. Clinicians are motivated to use tNGS because, compared with conventional methods, it provides a more complete resistance profiling and a much shorter time to results.

So far, I still compare it (the tNGS result) with the clinical condition, and it seems tNGS is very helpful in situations where a dispute cannot be resolved by other examinations. (CL 1, pulmonologist, individual interview)

### tNGS endorsement through the health professional network

Successful application of tNGS complex cases turned some clinicians into early adopters who recommended tNGS to other clinicians. This led to external referral from outside the programmatic network.

For example, I once encountered a problematic case from a doctor at another hospital. I then suggested they get a tNGS test. So, I advised them to send the sample to our hospital. (CL 1, pulmonologist, individual interview)

The clinicians who used tNGS are those who are the members of tNGS’s research network. By participating in this network, they could request tNGS to aid their clinical decision-making, with some even pursuing repeat testing should the result appear to be inconclusive.

We use it for problematic cases. Since this (tNGS) is still in the research phase and not widely available. So usually, we ask for help from a doctor [a researcher from tNGS implementation study] for tNGS test to help us with complicated cases. (CL 2, pulmonologist, individual interview)

### Lack of tNGS inclusion for patient management

The principal barrier in using tNGS in clinicians’ routine care is the absence of tNGS in the national DR-TB diagnostic algorithm and its relevant technical guidelines. Despite confidence in the result’s accuracy and being aware that tNGS has been recommended by the WHO, some of the clinicians felt legally prohibited from deviating from the current standard of care. This created a practice gap where clinicians perceived tNGS as a valuable tool for resolving complicated cases, but they could not formally act on its results.

Actually, I trust tNGS (result), but the technical guidelines don’t allow it, right? Our guidelines don’t permit it. For example, if there’s a (test) discrepancy, an additional resistance to, say, bedaquiline shown by tNGS, but the pDST still shows it as sensitive, the current guidelines do not allow us to act on that. (CL 4, pulmonologist, individual interview)

### Scarcity of local evidence to inform tNGS implementation policy

Despite the availability of WHO’s recommendation on tNGS use, our informants mentioned that inclusion of tNGS in the national DR-TB management guidelines would require laboratory technical guidance developed from Indonesian data. The NTP would also require cost-effectiveness analysis. The absence of such evidence has significantly slowed the programmatic adoption of tNGS in Indonesia.

The challenge is, once again, that we have not yet managed to obtain the good result for the (laboratory) step, which is crucial. It’s to know how we can get the best tNGS reading results. That is the biggest obstacle. And, again, why we need it? It’s because the technical guidelines must be very clear, for example, how many repetitions (tests) are allowed. (PM 1, former NTP official, individual interview)

So, we have to follow the (laboratory) evidence. After that, we can determine a cost-effective and cost-benefit strategy. That is very important for us in the Program (NTP). Our recommendation has to be based on the cost-effectiveness and cost-benefit. Meaning that we have to obtain the maximum possible benefit with the minimal cost. Therefore, we must establish the workflow steps for tNGS that are the most cost-effective and provide the greatest cost-benefit. (PM 1, former NTP official, individual interview)

## Discussion

tNGS has known advantages, but its implementation in high burden settings like Indonesia remains challenging. To date, Indonesia has not adopted tNGS as a culture-free method to diagnose DR-TB. Our findings highlighted that tNGS programmatic integration requires investment in human resources and a strengthened laboratory infrastructure. Furthermore, scaling up tNGS requires strong clinical engagement, which depends on the inclusion of tNGS in the DR-TB guidelines. However, this inclusion hinges on established local evidence such as diagnostic performance, cost-effectiveness analysis and clinical benefit.

Our study identified several technical barriers stemming primarily from the limited ease of use of tNGS when implemented in a programmatic setting, which consequently increases the overall operational burden. In the context of tNGS implementation, this perceived effort manifests in two critical ways: first, the tNGS workflow is technically complex, as echoed in other studies^18–20^; second, local workflow needs optimisation, as supported by other multicountry studies which reported that tNGS workflow is sophisticated and involves intricate procedures and significant investment in resources to tailor the workflow to their specific variables and needs.^3 20 21^ Bypassing labour-intensive tNGS optimisation steps risks sequencing failures and wasting resources.

With respect to the availability of highly skilled technicians with prior sequencing experience, we found that some of these highly skilled technicians were part of the previous COVID-19 response’s investments. This confirms suggestions by previous studies that COVID-19 era investments in diagnostic infrastructure and human resources could be advantageous for DR-TB programmes.^22 23^ In resource-limited settings, experienced technicians can also be leveraged to provide capacity-building training for newly recruited, less experienced staff. This finding aligns with other studies highlighting how peer mentoring can effectively increase human resource capacity in healthcare.^24–27^

With respect to clinical utility, tNGS has potential value for resolving complicated DR-TB cases, but this benefit was underused due to unclear tNGS position in national DR-TB guidelines. Without further guidance, clinicians are left unsure of the appropriate clinical follow-up of tNGS results. This aligns with evidence from other countries on healthcare technology adoption, which highlight formal guidelines as a key facilitator. A Delphi survey in the USA on NGS adoption found that clinicians viewed formal guidelines to be a critical solution for standardising reporting and follow-up protocols, providing the necessary reassurance to adopt the technology.^28^ Similarly, a study from Germany identified that established clinical guidelines were critical for adoption of new technologies by clinicians.^29^

The programmatic integration of tNGS in Indonesia needs a national guideline on tNGS use, which likely will need more local evidence (eg, laboratory data, clinical utility and cost-effectiveness) before adoption can be expected. However, developing technical guidelines is particularly challenging, as the established workflow should be tailored to different tNGS platforms and applicable nationwide. Currently, Indonesia is piloting the use of different types of tNGS for surveillance purposes, expecting that use of tNGS in clinical practice will be feasible in upcoming years when more data are available. This need is not unique; a multicountry implementation study echoed that local evidence on laboratory data, clinical impact and cost-benefit is perceived as critical for securing stakeholder buy-in and enabling wider adoption.^12^ These are a necessary step from a programmatic perspective. Given that tNGS entails a significant and recurring investment, generating local implementation evidence is crucial for ensuring that its integration is both evidence-informed and financially justifiable.

This study’s primary strength is its multistakeholder triangulation design, which captures a holistic view of health system readiness by integrating perspectives from clinicians, laboratory technicians and policymakers navigating a highly sophisticated technology. However, several limitations should be acknowledged. First, our findings are based on the Illumina Deeplex-MycTB assay, which may limit our findings to this platform, especially from laboratory aspects. Second, this study was also limited to West Java, noting that out of the 16 programmatic pDST culture laboratories nationwide, none except the West Java Provincial Health Laboratory currently possess operational sequencing capabilities. Therefore, our findings could not capture the distinct infrastructure and logistical constraints faced by other provinces, highlighting the need for expanded, nationwide feasibility studies as the technology scales.

## Conclusion

This exploratory qualitative study examined the perspectives of tNGS users, namely clinicians, laboratory personnel and policymakers. Our findings suggest that scaling up tNGS implementation for DR-TB programmatic adoption is perceived as operationally feasible, with some clinicians already incorporating its findings into clinical decision-making. However, scale-up will require a major effort and more skilled technicians to manage tNGS complexity. Additionally, scaling up this technology demands collaborative efforts among diverse stakeholders to formulate clear guidelines and resolve the ambiguity surrounding tNGS-based patient management. Consequently, the critical next step to transform tNGS into an integrated public health tool to manage DR-TB is to generate the necessary local evidence (in laboratory, clinical and cost-effectiveness studies), and work towards national DR-TB guidelines and implementation protocols.

## Supplementary material

10.1136/bmjopen-2026-117319 online supplemental file 1

10.1136/bmjopen-2026-117319 online supplemental file 2

10.1136/bmjopen-2026-117319 online supplemental file 3

10.1136/bmjopen-2026-117319 online supplemental file 4

## Data Availability

Data are available upon reasonable request.
