# Supplementary material for: Is targeted next generation sequencing for drug-resistant tuberculosis feasible in a high-burden setting: a qualitative study from Indonesia
Source: BMJ Open. 2026 Aug 11;16(8):e117319. doi: 10.1136/bmjopen-2026-117319 (PMC13475274; doi:10.1136/bmjopen-2026-117319)
Supplement: Supplementary file 2 [file bmjopen-16-8-s002.pdf]

Original study protocol

1. **Research aim**
   To identify the perceived barriers and enablers to the implementation of tNGS (targeted next-generation sequencing) for DR TB diagnosis in West Java, Indonesia.

2. **Methodology**
   a. Design: A qualitative study employing semi-structured interviews and focus group discussions (FGDs).
   b. Analysis: Data will be analyzed using a hybrid inductive-deductive approach. Initial coding will be conducted inductively from the raw transcript data. These codes will then be restructured through the lens of the healthcare acceptability framework. Final themes will be generated through this analytical integration and refined via iterative discussion among the research team.
   c. Participants & Sampling: Purposive sampling of key stakeholders (e.g., clinicians, lab technicians, policymakers) involved in TB care and control, and tNGS implementation.
   d. Data Collection: semi-structured interviews and FGDs will be conducted in-person or online (adjusting to participant preference and availability), audio-recorded with consent, and transcribed verbatim in Bahasa Indonesia.

3. **Data collection instruments**
   The instruments for individual interviews and FGDs are provided on the next page. The instruments are divided according to the participant's role.

4. **Ethics**
   Ethical approval for this study protocol was granted by the Health Research Ethics Committee at the Faculty of Medicine, Universitas Padjadjaran (Reference: 577/UN6.KEP/EC/2025).

Interview Guides for Clinicians

Background

1. How long have you been involved in MDR-TB management at the hospital?
2. What is your opinion on the landscape of second-line DST within the current program? Are there any shortcomings/areas for improvement?

Knowledge and Awareness

1. How familiar are you with tNGS technology?
2. Have you had any exposure to information about tNGS for DST? For example, through conferences, seminars, training, etc.
3. If you have had exposure, what is your opinion on the utilization of tNGS for DST?

Experience with tNG

1. Currently, WHO recommends tNGS as a test equivalent to pDST, but tNGS is not yet included in the national TB guidelines. Given this situation, how confident are you in the accuracy/validity of tNGS for making clinical decisions?
    a. Have you ever felt confident in the results but hesitant to use them in clinical decisions? Explain.
2. How do you use tNGS results?
    a. How do you interpret tNGS results?
    b. How often do you use tNGS results in clinical decision-making?
3. How easy is it to interpret tNGS results?
    a. How easy to understand is the language and format of the tNGS examination results prepared by Labkes Jabar?
    b. In your opinion, does the content of the tNGS results report meet expectations?
    c. Have you ever faced challenges in interpreting tNGS results?
    d. What is the follow-up when tNGS results are not-detected/test failure?
4. If tNGS results differ from pDST, what is your perception?
    a. How does this affect clinical decisions?
5. In your opinion, is tNGS able to help you manage patients better? In which aspect(s)? And how does tNGS help in that aspect(s)?
    a. What is your perception of the speed of receiving tNGS results compared to pDST results?
    b. What is your perception of the speed of receiving tNGS results in the context of patient management?
6. How do you position tNGS for DST?
    a. In your opinion, what is the position of tNGS in the overall DR-TB patient management algorithm?
    b. What then should be the ideal dynamic between tNGS and pDST? Replacing or complementing?
7. Overall, to what extent is tNGS utilized in your clinical practice?
    a. Treatment regimen determination

    b. Clinical guidance
    c. Diagnostic confirmation
8. Overall, what is your view on the idea of using tNGS as part of DR-TB management?
9. If tNGS continues to be available, will you continue to use tNGS? What are your reasons for continuing to use/not using it?
10. Would you recommend the use of tNGS to other PMDT hospitals? What are your reasons for recommending/not recommending it?

Technology Perception and tNGS Implementation

1. In your opinion, what are the advantages of tNGS compared to other DST methods?
2. In your opinion, what are the disadvantages and risks of tNGS compared to other DST methods?
3. In your opinion, if in the future tNGS is to be integrated into the standard MDR-TB diagnostics in Indonesia, what challenges will arise and what strategies should be implemented to overcome these challenges?

Operational Challenges (For Field Health Workers)

1. Have you ever experienced operational challenges with tNGS?
2. Are there any operational aspects of tNGS that hinder the entire tNGS workflow?
3. How do you and your team respond to these challenges?
4. From the first time your Hospital was involved with tNGS until today, what operational challenges have you experienced? For example
    a. Coordination
    b. Sample shipment

Interview Guides for Laboratory Technicians

Background

1. Can you describe your role and responsibilities at the Regional Health Laboratory (Labkesda)?
2. Do you have prior experience with molecular techniques and/or sequencing?

Knowledge and Awareness of tNGS

1. How familiar are you with tNGS technology? In your opinion, what is the purpose of the tNGS technology you are using?
2. Can you explain the workflow of tNGS for MDR-TB diagnostics? Can you guide me from sample reception to result reporting?
3. (For microbiology personnel) What is your perception of tNGS as a DST tool?
4. (For microbiology personnel) What is your perception of tNGS being culture-based?

Experience with tNGS

1. Looking at the entire testing process (sample preparation, library preparation, sequencing, data analysis, and reporting), how easily can you follow that workflow with minimal assistance? (Minimal assistance: able to work independently without asking Deepplex and the laboratory supervisor; staff can still read manuals and ask colleagues)
2. Are there any tNGS workflow steps that you find particularly difficult/complex?
3. How important/beneficial do you view the pre-PCR Deeplex (pre-extraction) process is for the tNGS results?
4. How user-friendly are the tNGS platform components?
    a. PCR (optional)
    b. Illumina machine
    c. Deeplex software
5. How easy is it for you to interpret tNGS results?
6. How is the process of evaluating tNGS processes and results that do not meet expectations?
7. What is the evaluation process for when resistance profiling fails? (e.g., mid-workflow sample dropping, Deeplex run failure, runs but no detection)
    a. How do you feel in situations where some samples are dropped, fail to run, or are not detected (ND)
    b. What about when resistance profiling results are partial?
8. How does the tNGS workflow compare to pDST in terms of efficiency and speed?
9. Do you think tNGS produces diagnostic results faster than pDST?
10. How do you feel about the efficiency of tNGS in producing successful results in a single run?
11. In your opinion, what is the accuracy and reliability of tNGS results?
12. How confident are you in the (resistance profiling) results you produce?
13. Overall, what is your view on the idea of using tNGS as part of routine laboratory program practice?

Perception of tNGS Feasibility

1. In your opinion, what are the advantages of tNGS compared to pDST?
2. In your opinion, what are the disadvantages and risks of tNGS compared to pDST?
3. Have you ever experienced operational management challenges with tNGS?
4. Are there any aspects of tNGS operational management that hinder the entire workflow? (e.g., workflow coordination, inter-facility coordination, specimen transport)
5. How do you and your team respond to these challenges?
6. From August until today, what sustainability and operational management challenges have you faced? For example:
    a. Human resources
    b. Consumables
    c. Equipment maintenance
7. In your opinion, if tNGS is to be integrated into the standard MDR-TB diagnostics in Indonesia in the future, what challenges will arise and what strategies should be implemented to overcome them?
    a. Is the training sufficient? What could be improved?
    b. What was not covered in the training that should be included?
    c. What can be done to retain skills?

Suggestions and Recommendations on Training

1. What is your feedback on the training that has been provided?
    a. What aspects could be What aspects were not discussed in the training?
    b. What do you usually do to maintain the skills and knowledge acquired during the training?

Interview Guides for Policymakers

Perception of tNGS Technology

1. In your opinion, what are the advantages and disadvantages of tNGS compared to other MDR-TB diagnostic tools (e.g., Xpert MTB/RIF, culture)?
2. In your opinion, how well does the implementation of tNGS align with the National TB Strategic
3. Do you see tNGS as a vital tool for achieving national TB control goals? Which specific goals could be achieved through the implementation of tNGS?
4. In your future view, will tNGS complement or replace currently commonly used diagnostic methods, namely pDST/Xpert Ultra?

Perception of Financing and Cost-Effectiveness

1. In your opinion, if tNGS were to be adopted, what would be the funding mechanism/scheme for its implementation? (e.g., government funding from the National/Regional Budget, mixed financing with external donors like the Global Fund)
2. In your opinion, if tNGS is adopted, how sustainable would it be in the long term, considering its maintenance and operational costs?
3. Do you have any concerns regarding the costs and funding for tNGS implementation?
4. In your opinion, will the costs to be incurred for implementing tNGS be commensurate with the benefits offered by the technology?

Perception of the tNGS Implementation Process

1. In your opinion, what is the main advantage of tNGS that would be the primary motivation for the Ministry of Health to adopt it?
    a. To optimize MDR-TB treatment in terms of regimen accuracy, leading to improved treatment outcomes
    b. To help achieve TB control goals in Indonesia.
2. In your opinion, how will the demand from global/national/local stakeholders influence the decision to adopt tNGS?
3. In your opinion, does Indonesia's current health system have adequate infrastructure (laboratory, IT systems) to support tNGS implementation?
    a. What health system components are still inadequate?
4. In your opinion, what policies/regulations could facilitate or hinder the adoption of tNGS? (e.g., BPJS/JKN - National Health Insurance)
5. In your opinion, what are the challenges to adopting tNGS into routine MDR-TB diagnostics?
    a. Implementation costs
    b. Funding mechanisms and availability
    c. Infrastructure readiness
    d. Policies/regulations
6. Overall, in your opinion, how feasible is it to adopt tNGS as a routine MDR-TB diagnostic tool in Indonesia?
7. How do you feel about this process? Do you have any concerns?
