# Supplementary material for: Is targeted next generation sequencing for drug-resistant tuberculosis feasible in a high-burden setting: a qualitative study from Indonesia
Source: BMJ Open. 2026 Aug 11;16(8):e117319. doi: 10.1136/bmjopen-2026-117319 (PMC13475274; doi:10.1136/bmjopen-2026-117319)
Supplement: Supplementary file 3 [file bmjopen-16-8-s003.pdf]

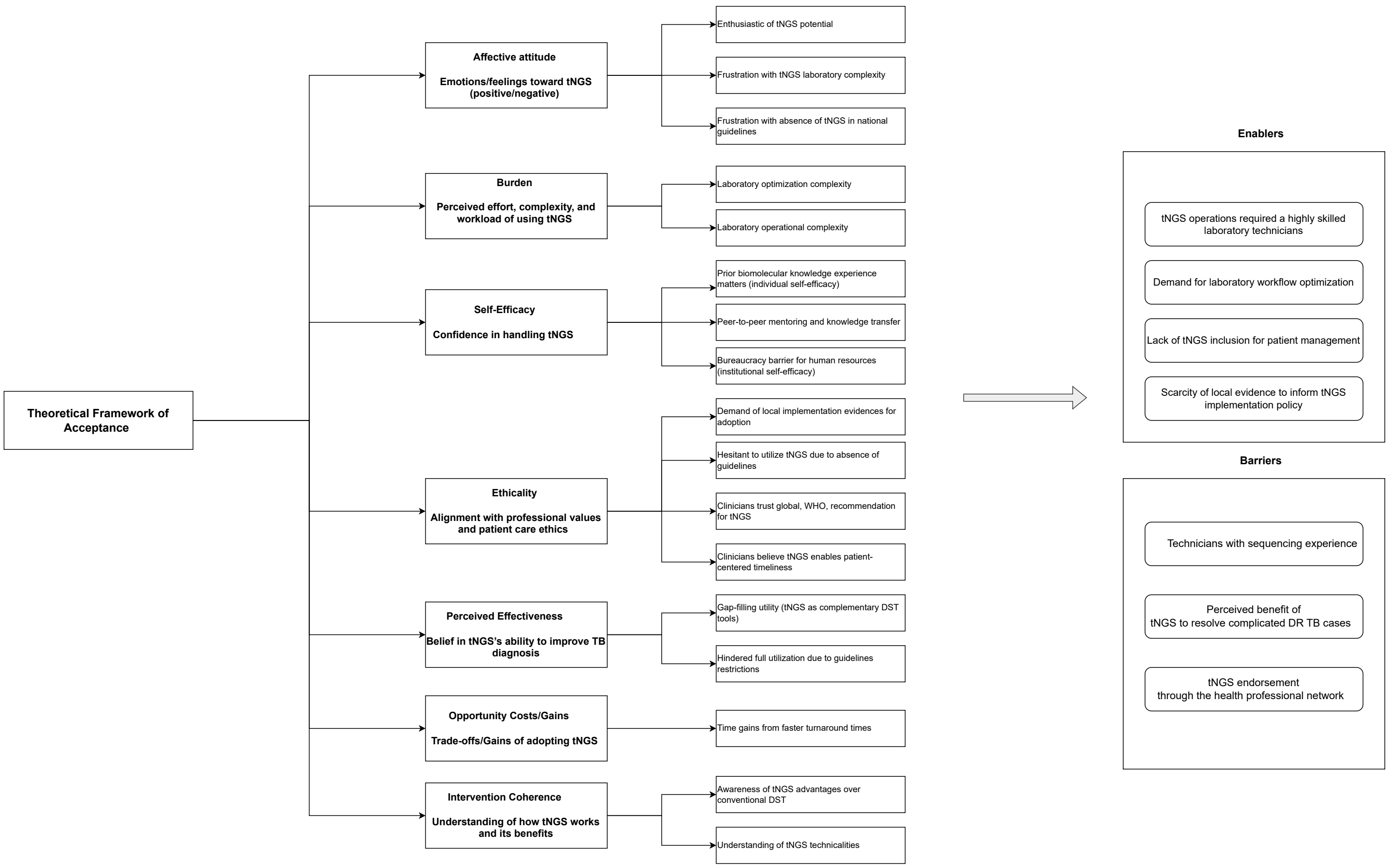
Theoretical Framework of Acceptance
Affective attitude
Emotions/feelings toward tNGS (positive/negative)
Enthusiastic of tNGS potential
Frustration with tNGS laboratory complexity
Frustration with absence of tNGS in national guidelines
Burden
Perceived effort, complexity, and workload of using tNGS
Laboratory optimization complexity
Laboratory operational complexity
Self-Efficacy
Confidence in handling tNGS
Prior biomolecular knowledge experience matters (individual self-efficacy)
Peer-to-peer mentoring and knowledge transfer
Bureaucracy barrier for human resources (institutional self-efficacy)
Ethicality
Alignment with professional values and patient care ethics
Demand of local implementation evidences for adoption
Hesitant to utilize tNGS due to absence of guidelines
Clinicians trust global, WHO, recommendation for tNGS
Clinicians believe tNGS enables patient-centered timeliness
Perceived Effectiveness
Belief in tNGS's ability to improve TB diagnosis
Gap-filling utility (tNGS as complementary DST tools)
Hindered full utilization due to guidelines restrictions
Opportunity Costs/Gains
Trade-offs/Gains of adopting tNGS
Time gains from faster turnaround times
Intervention Coherence
Understanding of how tNGS works and its benefits
Awareness of tNGS advantages over conventional DST
Understanding of tNGS technicalities
Enablers
tNGS operations required a highly skilled laboratory technicians
Demand for laboratory workflow optimization
Lack of tNGS inclusion for patient management
Scarcity of local evidence to inform tNGS implementation policy
Barriers
Technicians with sequencing experience
Perceived benefit of tNGS to resolve complicated DR TB cases
tNGS endorsement through the health professional network
