# Supplementary material for: Is targeted next generation sequencing for drug-resistant tuberculosis feasible in a high-burden setting: a qualitative study from Indonesia
Source: BMJ Open. 2026 Aug 11;16(8):e117319. doi: 10.1136/bmjopen-2026-117319 (PMC13475274; doi:10.1136/bmjopen-2026-117319)
Supplement: Supplementary file 4 [file bmjopen-16-8-s004.pdf]

Is targeted Next Generation Sequencing for Tuberculosis feasible in a high-burden TB setting: a qualitative study from Indonesia

Table of Illustration quotes

TFA constructs	Codes	Illustration quotes
Affective attitude	Enthusiastic about tNGS potential	"The absence of this tool (tNGS) in our service... we cannot say it hinders us; it cannot be a hindrance. We cannot claim that. Why? Because our standard, our protocol, has already been established by the Ministry of Health. However, the presence of this tool presents an opportunity to reach a (clinical) decision more quickly." (CL 6, clinical pathologist, individual interview)
		"Although the current TCM XDR is quite helpful, it is still, after all, a rapid test. This (tNGS), however, is more than just a rapid tool. I mean, it would certainly be even more helpful If, for instance, it (tNGS) could become the standard guideline for all patients, that would be of great assistance for patients." (CL 2, pulmonologist, individual interview)
	Frustration of the absence of tNGS in the national TB guidelines	“As long as there is no official policy (for tNGS), it’s understandable that we (clinicians) are somewhat hesitant to make a definitive statement (whether tNGS could be helpful or not). But for me, I would—let me be clear, for me personally, I would certainly choose tNGS (if compared with pDST). But as mentioned, I don’t know the decision regarding that (official tNGS policy). So I’m remaining unsure." (CL 4, pulmonologist, individual interview)
	Frustration with the tNGS laboratory complexity	"Here is an example: in the early days, we always held the view that with a high concentration, we would obtain a good result. But currently, when we obtain a very high concentration and it does not yield the expected or good result. that raises a new question. So, what exactly is wrong with the workflow protocol? If we look at the measurement method used, for instance, for concentration using Qubit, it is indeed theoretically correct why that method is employed. And we are not actually mistaken in using it." (LM 1, laboratory manager, individual interview)
		"Each stage (of tNGS) also contributes because the longer the process, the more difficult it is to break down where our error lies. So, if the stages are very long, it becomes increasingly difficult for us to troubleshoot, to pinpoint where such a result actually came from. It's

		different if, for example, we use GeneXpert, you just dilute it and then load it. We already know, right, that means the issue is only in this part. But if the process is very long, we have difficulty troubleshooting exactly where the problem is." (TO 1, tNGS operator, individual interview)
Burden	Laboratory optimization complexity	"Back then, we had no way to see the inhibitor. The only option was to test the reagents first... to find which sequencing reagent produced the best result. However, conducting that trial requires both money and time. That's why identifying the inhibitor was quite (technically and financially) challenging." (LM 1, laboratory manager, individual interview)
		"From my personal perspective, the future addition of a new technical method will certainly create… how to put it? Like additional time requirements for us, especially for optimization, the workflow has to adapt to the new method. Because it (new method) add an extra step, right? So we have to figuring out the most efficient procedure. That's probably where the main challenge lies. So, it requires time for adaptation." (TO 4, tNGS operator, FGD)
	Laboratory operational complexity	"Because it is indeed so complex, it is somewhat difficult to troubleshoot problems since there are genuinely many stages. For example, with PCR, it's just extraction, PCR, and then the result comes out. With this (tNGS), it's extraction, library prep. Within library prep alone there are several stages. We can have up to five or six stages." (TO 1, tNGS operator, individual interview)
		"If we're talking about complexity, it could be said to be in the library prep stage. Because there you have concentration calculations, then normalizing concentrations, and also pipetting with high accuracy like pipetting one microliter, two microliters. That is perhaps the most challenging part." (LM 1, laboratory manager, individual interview)
Self-Efficacy	Prior biomolecular knowledge and experience	"I think the most important is experience. It's risky (to rely on inexperienced technicians). For example, you need to understand the theory behind tNGS, like how PCR works, how library preparation works, and what single-stranded DNA is. Then, how does (nucleic acids) amplification work? These things require knowledge and experience. If they don't have these, then it will be a bit difficult. They could get stuck." (LM 1, laboratory manager, individual interview)

		“In my view, if the context is that this (tNGS) will be upscaled, I believe there are certain requirements that are essential. However, right now, I know that there are also constraints; for instance, it’s not easy to find people who have experience with sequencing, who must also be proficient in bioinformatics, like that.” (TO 5, tNGS operator, FGD)
	Peer-to-peer mentoring and knowledge transfer	"So for us, it's not that we are already experts in doing this; rather, we are also learning together, teaching each other, in order to be able to carry out this (sequencing)." (TO 8, tNGS operator, FGD)
		"Speaking from my experience… when I first started, I genuinely didn't know anything about tNGS, so I just learned from [other tNGS technicians]. So, I mostly learned from her and read on my own." (TO 4, tNGS operator, FGD)
	Bureaucracy barrier for human resources (institutional self-efficacy)	“For it (tNGS) to become a routine program, in my opinion, the changes would have to be very large-scale. The change even started from the recruitment system. It has to be fixed first. So, we have to open recruitment for those with a molecular biology background. But several labs can't just recruit technicians freely on demand.” (TO 1, tNGS operator, individual interview)
Ethicality	Demand of local implementation evidences for adoption	"That’s what I need is data. Its (tNGS) diagnostic value. That’s really the main thing. Later, for instance, if its diagnostic value is already proven to be good, and then perhaps it will show… what are the strengths of tNGS in terms of drugs, and what are those of LPA." (CL 1, pulmonologist, individual interview)
	Hesitant to utilize tNGS due to absence of guidelines	"However, to my knowledge, there is not yet any directive stating that we may bypass the official (TB) guidelines, none yet, I think. I will have to inquire about that later. Perhaps due to my workload, I am not fully informed on that matter." (CL 4, pulmonologist, individual interview)
		“If, for example, in the future we can make it (tNGS) into policy that is included in the official guidelines, then perhaps that will become our new way of speeding up the process of identifying resistance patterns. But for now, I can’t really do anything.” (CL 3, internist, individual interview)
	Clinicians trust global WHO recommendation for tNGS	"Because it is rapid and accurate. I have read the systematic review and meta-analysis; the WHO has already published it, so I trust it and will use it to inform my decisions. Besides, even within the research

		framework, the tool is available, and we can utilize it for our patients. Moreover, the WHO has already recommended it." (CL 2, pulmonologist, individual interview)
	Clinicians believe tNGS enables patient- centered timeliness	"Previously, if we waited for pDST, it took 2-3 months. During that 2-3 month period, it is not counted, not considered as active treatment. However, if we can shorten it through tNGS, for instance, especially if it can be just one or two weeks, then the time the patient is considered off medication is only those two weeks. After that, we can start again with a regimen better suited to the patient, so that the patient doesn't waste time." (CL 5, pulmonologist, individual interview)
		"Furthermore, the second issue is the lengthy time to obtain pDST results, whereas therapeutic decisions must be made immediately." (CL 1, pulmonologist, individual interview)
Perceived Effectiveness	Gap-filling utility (tNGS as complementary DST tools)	"So, nothing is perfect. Therefore, my hope is that the two (pDST and tNGS) will complement each other. That is precisely why I don't wish for that (pDST) to be discarded, but rather for its role to shift (to become confirmatory diagnostic). This is related to what? It is related to the speed of decision-making in initiating therapy." (CL 2, pulmonologist, individual interview)
		"If what it (tNGS) can offer is that tNGS can provide more precise treatment, then it should certainly be able to replace XDR, right? Right from the start. Just like that. It can later be evaluated against the results from pDST." (CL 7, clinical microbiologist, individual interview)
	Hindered full utilization due to the guidelines restrictions	"Because the WHO positions it as a first-line method equivalent to pDST, with its rapid decisions and so forth. However, currently, since it's (tNGS) not yet official here, we still use it as a second-line tool. So through this we use it when there is a discrepancy here. Like between pDST results and the clinical condition. So what will determine the decision is tNGS. tNGS serves as the third opinion." (CL 2, pulmonologist, individual interview)
		"We still monitor the patient, of course. If the guidelines require a specific action, then that's that. However, we continue to observe the patient first. Now, as it turned out recently, there was a new discrepancy: there was this one patient, a young adult. The issue is that the guidelines do not permit that approach (tNGS), so I opted for close observation instead, very close observation." (CL 4, pulmonologist, individual interview)

Intervention Coherence	Awareness of tNGS advantages over conventional DST	"Actually, regarding tNGS, it stands in a middle position, right? Based on my understanding, it's quite fast like some test can be obtained in just 8 or 9 days. Yes, there are indeed cases that take longer, perhaps due to various reasons like waiting in the queue or delays in communicating results, which can prolong the turnaround time. But mostly, before DST results are available, the tNGS result is usually already there." (CL 5, pulmonologist, individual interview)
		"I mean, of course, this one (tNGS) is certainly faster. Phenotypic (testing) takes a long time. I feel bad for the patient." (CL 1, pulmonologist, individual interview)
	Understanding of tNGS technicalities	"In its fundamentals, it's indeed a method that can examine the bacteria comprehensively, not the patient, but the bacteria themselves. And if there are constraints with a sample that is not capable, that is more about how we optimize the pre-analytical phase. It's not about judging the method itself (tNGS) as being unsuitable. We simply need to optimize, acknowledging that here the quality of the specimen to be examined may be insufficient, and we must improve on that front." (LM 1, laboratory manager, individual interview)
		"It is somewhat difficult, honestly, because we cannot guarantee the quality of the sample itself. Even though we can guarantee that we can produce a result within 7 days, we don't know what the quality of the patient's sample is like. That is what is most critical for sequencing. We can work on it for a week, a whole week, but the result could still be many ND (not detected). That result cannot be used." (TO 1, tNGS operator, individual interview)
Opportunity Costs/Gains	Time gains from faster turnaround times	"pDST has its own advantage capable of observing, for observing the bacteria that are truly active within the human body, and there, it identifies where the drug resistance lies. However, when it comes to speed, that is its drawback. It's very, very slow compared to tNGS." (LM 1, laboratory manager, individual interview)
		"That advantage is a matter of time, it's (tNGS) faster, and also accurate. As for accuracy, it's the same with (phenotypic) DST, right? But based on my experience, what helps the most is the time saved." (CL 2, pulmonologist, individual interview)
