# Supplementary material for: Is targeted next generation sequencing for drug-resistant tuberculosis feasible in a high-burden setting: a qualitative study from Indonesia
Source: BMJ Open. 2026 Aug 11;16(8):e117319. doi: 10.1136/bmjopen-2026-117319 (PMC13475274; doi:10.1136/bmjopen-2026-117319)
Supplement: Supplementary file 5 [file bmjopen-2026-117319.reviewer_comments.pdf]

PEER REVIEW HISTORY

BMJ Open publishes all reviews undertaken for accepted manuscripts. Reviewers are asked to complete a checklist review form and are provided with free text boxes to elaborate on their assessment. These free text comments are reproduced below.

ARTICLE DETAILS

Title (Provisional)

Is targeted Next Generation Sequencing for drug-resistant tuberculosis feasible in a high-burden setting: a qualitative study from Indonesia

Authors

Annisa, Salma N; Alifia, Almira; Larasmanah, Alamanda N; Ramadhani, Nurlaila S; Indriani, Silvi; Soeroto, Arto; van Crevel, Reinout; Hill, Philip; Lestari, Bony Wiem

---

VERSION 1 - REVIEW

**Reviewer**	**1**
**Name**	**Watson , Basilea**
**Affiliation**	**National Institute of Research in Tuberculosis**
**Date**	**24-Feb-2026**
**COI**	**I confirm that I have previously co-authored publications with Phillip C Hill. I have no other competing interests to declare.**

The abstract contains grammatical errors and several unclear phrases (e.g., “lack of evidences,” “required a coordinated,” “include it into national policy”). The conclusion slightly overstates feasibility (“technically feasible”) without clearly qualifying the context as exploratory qualitative findings. Minor language editing and tightening are needed for clarity and balance.

**Reviewer**	**2**
**Name**	**Seas, Carlos**
**Affiliation**	**Universidad Peruana Cayetano Heredia**
**Date**	**02-Mar-2026**
**COI**	**None**

The authors should be commended for conducting a qualitative study aimed at identifying the barriers to implementing tNGS for DR-TB diagnosis in West Jav, a highly endemic area of

TB in Indonesia. While the results are very interesting, this reviewer has some concerns as follows:

1. What is the external validity of your results for the rest of Indonesia?

2. How interesting are these results to a broader audience?

3. Aren't these results predictable?. Did you experience the same responses when implementing Xpert MTB/RIF?

4. Why were the guides not subject to a pilot study?

5. I would like to see the study design's weaknesses and strengths well identified in the discussion section.

**Reviewer**	**3**
**Name**	**GUIDO, GIACOMO**
**Affiliation**	**University of Bari Aldo Moro**
**Date**	**26-Apr-2026**
**COI**	**None**

---

1. The use of purposive sampling is appropriate, but the manuscript does not clearly justify how participants were selected beyond general roles and how diversity of perspectives (especially policymakers) was ensured. The very limited number of policymakers may weaken the credibility of system-level conclusions. I think this should either be expanded or clearly reframed as exploratory.

2. The authors state that "thematic saturation was reached," but no description of how saturation was assessed. At the same time, there is no indication of iterative stopping criteria. Given the small sample and heterogeneity of stakeholders, saturation is questionable and needs explicit methodological support

3. In this work, participants were recruited from an ongoing implementation study. Hence, we could imagine that participants were already exposed to or engaged with tNGS, predisposing toward favorable perceptions. This introduces systematic bias, particularly in reported "enablers," and should be explicitly acknowledged

4. Several minor issues reduce readability. There are some grammatical inconsistencies (e.g., "lack of evidences," should be lack of evidence, "required a coordinated" should be "requires a coordinated"; "absence tNGS formal endorsement" could be better as "the absence of formal tNGS endorsement"), occasional repetition and long sentences.

VERSION 1 - AUTHOR RESPONSE

	**Reviewer 1**	
1	The abstract contains grammatical errors and several unclear phrases (e.g., "lack of evidence," "required a coordinated," "include it into national policy").	Thank you for this comment. We have thoroughly proofread the entire abstract and revised it accordingly. Specifically, we have replaced the ambiguous phrases highlighted in your comment. Changes can be found in lines 51, 82-83, and 85-86.
2	The conclusion slightly overstates feasibility ("technically feasible") without clearly qualifying the context as exploratory qualitative findings.	We agree with the reviewer that our original phrasing overstated technical feasibility without properly framing it within the bounds of an exploratory qualitative inquiry. We have revised the conclusion to avoid overstatement. Changes can be seen in lines 456-458.
3	Minor language editing and tightening are needed for clarity and balance.	Thank you for this constructive feedback. We have thoroughly proofread the manuscript and polished the language to improve clarity, grammar, and narrative flow. This was completed with the close assistance of professional co-authors and colleagues who are proficient in written academic English.
	**Reviewer 2**	
1	The authors should be commended for conducting a qualitative study aimed at identifying the barriers to implementing tNGS for DR-TB diagnosis in West Java, a highly endemic area of TB in Indonesia. While the results are very interesting, this reviewer has some concerns as follows:	We sincerely thank the reviewer for the encouraging words and for recognizing the value of conducting this qualitative inquiry within West Java.
2	What is the external validity of your results for the rest of Indonesia?	We thank the reviewer for highlighting the important issue of generalizability. We have updated our limitations section to provide crucial country-level context that explains the current boundaries of sequencing in Indonesia. Changes can be seen 443-452.
3	How interesting are these results to a broader audience?	Thank you for this comment. Following the 2023 WHO guidelines recommending tNGS (WHO, 2023), empirical data on how local clinicians and lab systems actually adapt to this technology remain scarce. To address we have included a brief elaboration on the gap of this study, which can be seen in lines 130-138.

		In the Discussion section, we also discussed key barriers/enablers that will also provide insights for other countries aiming to implement tNGS in lines 393-399.
4	Aren't these results predictable? Did you experience the same responses when implementing Xpert MTB/RIF?	We appreciate this critical perspective. We agree that certain health system barriers, such as initial funding sustainability and updated national guidelines, are common across any new diagnostic implementation, as we found in our Xpert evaluation studies (Nijman et al., 2026) https://link.springer.com/article/10.1186/s12913-026-14658-0.<br><br>In this study, we found the operational aspects of tNGS are distinct from Xpert. Xpert MTB/RIF is a low-complexity molecular assay designed for automated, point-of-care utility. In contrast, tNGS is a high-complexity assay requiring specialized reference laboratory networks, intricate manual library preparation, in-house workflow optimizations, and advanced training for technicians and clinicians alike.
5	Why were the guides not subject to a pilot study?	Thank you for raising this point regarding tool validation. We agree that piloting is ideal; however, because our eligible informant pool was highly specialized and limited, conducting a formal pilot study would have significantly depleted our available sample size for the primary analysis. We have updated this part, and changes can be seen in lines 206-208.
6	I would like to see the study design's weaknesses and strengths well identified in the discussion section.	We thank the reviewer for the comment. We have thoroughly revised and expanded the study design, weaknesses, and strengths at the end of the discussion section, which can be seen in lines 443-452.
	**Reviewer 3**	
1	The use of purposive sampling is appropriate, but the manuscript does not clearly justify how participants were selected beyond general roles and how diversity of perspectives (especially policymakers) was ensured. The very limited number of policymakers may weaken	Thank you for your comment. We are aware that the number of policymakers included was very limited due to the very early stage of tNGS use for DR-TB in Indonesia. Following your advice, we have explicitly mentioned this study as exploratory as suggested in lines 57, lines 148, and 168. We also added a brief explanation in lines 142-145.

	the credibility of system-level conclusions. I think this should either be expanded or clearly reframed as exploratory.	
2	The authors state that “thematic saturation was reached,” but no description of how saturation was assessed. At the same time, there is no indication of iterative stopping criteria. Given the small sample and heterogeneity of stakeholders, saturation is questionable and needs explicit methodological support	Thank you for your comment. We have added the description of how saturation was assessed and determined in lines 215-217.
3	In this work, participants were recruited from an ongoing implementation study. Hence, we could imagine that participants were already exposed to or engaged with tNGS, predisposing toward favorable perceptions. This introduces systematic bias, particularly in reported “enablers,” and should be explicitly acknowledged	Thank you for your thoughtful comment. Given that tNGS is a novel and complex technology for DR-TB use in our setting, this study purposefully focused on individuals directly exposed to its utilization and management to answer our objectives. Therefore, not including individuals without tNGS exposure was a deliberate boundary of our study design. However, we acknowledge that individuals not exposed to tNGS would have different perspectives that are valuable and represent a critical next step, and we have added elaboration on this in lines 98-100.
4	Several minor issues reduce readability. There are some grammatical inconsistencies (e.g., “lack of evidences,” should be lack of evidence, “required a coordinated” should be "requires a coordinated"; "absence tNGS formal endorsement” could be better as “the absence of formal tNGS endorsement”), occasional repetition and long sentences.	Thank you for pointing out these issues. We have thoroughly proofread the manuscript to improve overall readability and correct the grammatical inconsistencies. In response to your specific comments, we have also revised our terminology in lines 51 and 82-83.
